# Stanniocalcin-1 Overexpression Prevents Depression-Like Behaviors Through Inhibition of the ROS/NF-κB Signaling Pathway

**DOI:** 10.3389/fpsyt.2021.644383

**Published:** 2021-06-14

**Authors:** Bin Chao, Lili Zhang, Juhua Pan, Ying Zhang, Yuxia Chen, Manman Xu, Shijing Huang

**Affiliations:** ^1^Traditional Chinese Medicine Research and Development Center, Guang'anmen Hospital, China Academy of Chinese Medicine, Beijing, China; ^2^Department of Endocrinology, Guang'anmen Hospital, China Academy of Chinese Medicine, Beijing, China

**Keywords:** depression, stanniocalcin-1, reactive oxygen species, NF-κB, inflammation, neural plasticity

## Abstract

**Background:** Depression is a burdensome psychiatric disorder presenting with disordered inflammation and neural plasticity. We conducted this study with an aim to explore the effect of stanniocalcin-1 (STC1) on inflammation and neuron injury in rats with depression-like behaviors.

**Methods:** A model of depression-like behaviors was established in Wistar rats by stress stimulation. Adeno-associated virus (AAV)-packaged STC1 overexpression sequence or siRNA against STC1 was introduced into rats to enhance or silence the STC1 expression. Moreover, we measured pro-inflammatory and anti-inflammatory proteins, superoxide dismutase (SOD), catalase (CAT), malondialdehyde (MDA) and reactive oxygen species (ROS) production. An *in vitro* model was induced in hippocampal neurons by CORT to explore the effect of STC1 on the neuron viability, toxicity and apoptosis. RT-qPCR and Western blot assay were employed to determine the expression of STC1 and nuclear factor κB (NF-κB) signaling pathway-related genes.

**Results:** STC1 was under-expressed in the hippocampus of rats with depression-like behaviors, while its overexpression could reduce the depression-like behaviors in the stress-stimulated rats. Furthermore, overexpression of STC1 resulted in enhanced neural plasticity, reduced release of pro-inflammatory proteins, elevated SOD and CAT and diminished MDA level in the hippocampus of rats with depression-like behaviors. Overexpressed STC1 blocked the ROS/NF-κB signaling pathway, thereby enhancing the viability of CORT-treated neurons while repressing their toxicity and apoptosis.

**Conclusion:** Collectively, overexpression of STC1 inhibits inflammation and protects neuron injury in rats with depression-like behaviors by inactivating the ROS/NF-κB signaling pathway.

## Introduction

Depression is regarded as a disabling chronic mental disease (1), and afflicts ~300 million individuals on a global scale (2). Depression is featured by inflammation and immune activation, in addition to autoimmune reactions against self-epitopes (3). The occurrence of depression is associated with the adverse life experiences, genetic variations, as well as environmental factors (4). Moreover, disrupted neural plasticity is closely related to the development of depression (5). Depression is prone to cause a huge burden to the patients by diminishing their life quality and threatening their somatic health; it has also been revealed to elevate the risk of other diseases such as stroke and diabetes (6). At present, antidepressants are recommended as the standard treatment for adult patients suffering from moderate to severe major depression, but the efficacy is still not satisfactory in some individuals (7). Against such backdrop, it is still important to seek novel target for treatment of depression.

Stanniocalcin-1 (STC1) is identified as a glycoprotein hormone that is able to regulate multiple biological processes including inflammation and oxidative responses (8). STC1 has been reported as an important regulator in the neuroprotection of human marrow-isolated adult multilineage inducible cells and human neural stem cells (9). Moreover, mammalian STC1 in brain neurons is able to protect neurons against hypercalcemic as well as hypoxic injury (10). Intriguingly, it has been reported that STC1 is capable of inactivating the reactive oxygen species (ROS)/nuclear factor κB (NF-κB) pathway in neural cells, thereby rescuing them from ischemic injury (11). Several NF-κB targets are essential for regulation of the ROS production, which in turn possesses inhibitory or promoting roles in the NF-κB signaling pathway (12). The NF-κB signaling pathway constitutes a target for anti-inflammatory drugs, as it represents a prototypical proinflammatory signaling pathway, largely depending on the role of NF-κB in the expression of proinflammatory genes including chemokines, cytokines and adhesion molecules (13). The NF-κB signaling pathway is thought to be crucial in the development of major depressive disorders (14). Importantly, the NF-κB signaling pathway regulates many genes related to inflammation as well as neural plasticity, hence affecting neurogenesis (15). Of note, ROS is considered responsible for a series of neural diseases such as traumatic brain and degenerative neural injuries and its generation in neural tissues is thus regarded as a typical marker for neural diseases (16). Additionally, generation of ROS could aid myeloid-derived suppressor cells to inhibit T-cell response in patients with major depression (17). Interestingly, STC1 could negatively mediate the level of ROS production in mesenchymal stromal cells from human palatine tonsil (18), as well as in lung cancer cells (19). In light of the above findings, this study (Supplementary Figure 1) was performed to validate a hypothesis that STC1 may mediate inflammation and neural plasticity in depression-like behaviors *via* the ROS/NF-κB signaling pathway.

## Methods

### Ethical Approval

The study was approved by the Animal Ethics Committee of Guang'anmen Hospital, China Academy of Chinese Medicine. All experimental procedures strictly obeyed the National Institutes of Health Animal Use and Care Guideline.

### Establishment of Rat Model With Depression-Like Behaviors

A total of 125 male Wistar rats weighting 160–180 g (SLAC Laboratory Animal Co., Ltd., Shanghai, China) were enrolled in this study. The rats were individually housed at room temperature of 22–24°C under illumination of 12-h light/dark cycles for 1 week before the experiment. In brief, the rats were exposed to stimulation of chronic unpredictable mild stress for 5 weeks, during which the rats were deprived of food and water for 24 h, respectively, and subjected to 45° cage tilt for 24 h, 5-min cold-water swimming at 4 °C, wet bedding for 24 h, foot electric shock at 0.5 mA for 0.5 s, physical restraint for 2 h, cage shaking for 2 h, and overnight illumination. The stress was performed to each rat randomly every day in unpredictable order (20). Five weeks later, behavior tests and biochemical tests were performed.

### Animal Grouping and Adeno-Associated Virus Infection

The normal rats were non-treated while the rats with depression-like behaviors were non-treated or given injection of AAV-packaged negative control sequence, STC1 overexpression (oe-STC1) sequence or small interfering RNA against STC1 (si-STC1) in the hippocampus CA1 region of rats. Rats were anesthetized with pentobarbital sodium (150 mg/kg, i.p.) and placed in a stereotactic frame. Using an electric micro-syringe pump (Stoelting, USA), inject 1.5 μL purified and concentrated AAV was injected at a speed of 150 nl/minute into the hippocampus CA1 region of rats (about 1,012 infection units/mL), (with the Bregma as the coordinate, −3.48 mm; medial/lateral, ± 1.8 mm; dorsal/ventral, −2.55 mm). Behavior experiments or biochemical tests were performed 10 days after injection. The injection site was verified by behavioral testing, and only rats with the correct injection site were used in the following experiments. Six serial sections (30 um) were selected in each CA1 region, and the infected cells were counted under a fluorescence microscope. The total number of neuron signals was averaged by counting the serial sections of 3 normal rats. The infection rate is expressed as the number of infected cells/total neurons (20). The AAV was purchased from Shanghai Genechem Co., Ltd. (Shanghai, China), while primer sequence, vector construction, package, and purification were made by Genechem. The AAV-packaged siRNA was constructed using the GV478 vector, the AAV-packaged overexpression plasmid was constructed using the GV467 vector.

The weight and food intake of rats were recorded after the experiment. The rats were weighed and provided food at 9:00 every day. They were weighed again at 9:00 the next day. Eight rats were selected from each group, and the average value was recorded.

### Sucrose Preference Test

During acclimatization, the rats were individually housed in a cage containing two bottles of sucrose solution (1%, w/v) for 24 h. During the second 24 h, a bottle of sucrose solution was replaced with a bottle of tap water. In the experimental phase, the rats were initially deprived of water and food for 24 h. After that, two bottles were placed into the cage for 3 h, one of which contained 100 mL of 1% sucrose solution and the other contained 100 mL of tap water. Sucrose preference is defined as sucrose consumption/[water consumption + sucrose consumption] × 100% in the 3-h test. Eight rats were selected from each group, and the average value was recorded (20).

### Forced-Swimming Test

The forced-swimming test was carried out at 13:00–15:00 P.M. at the 8th and 9th week. The experiment was conducted in a dark room and an illumination lamp (40 W) was used to keep the light unchanged in the laboratory. In this experiment, the rats were placed in an open cylindrical container (25 cm long and 16 cm high), and filled with 25 cm high water, with a temperature of about 23 ± 2°C. Using the single blind method, a group with more than 7 rats was considered to be with statistical significance. The time measured was immobility time (when there was only slight movement when the rats were floating on the water surface or when the body was perpendicular to the water surface and only the nose came out of the water), and upward struggling time (the vigorous movement of front claw grasping the glass wall). The immobility time and struggling time of rats in each group were tested; the total recording time was 360 s, and the data from the last 4 min were recorded with the software program (20).

### Open-Field Test

The rats were placed in a 100 × 100 × 40 cm opaque box, with bottom surface composed of 16 equal squares. In the dark rooms, the incandescent lamp (100 W) was turned on to focus on the filed about 110 cm above the ground, and the rats were placed in the center each time. The measurement time for each was 5 min, and the test process was recorded using a Video tracking software (Any-maze). Each rat was only tested once. After the measurement, the feces were cleaned up, and wiped using 75% alcohol before the next testing. Using the single-blind method, we counted the number of the lattices that the rats passed through (four claws passing through) within 5 min, and recorded the exercise time and the number of times of standing (two front claws leaving the bottom) with the software program. Each group contained 8 rats, and the average value was recorded (21).

### Tail Suspension Test

Tail suspension tests are used to test the variability of animal behaviors. Using the tail suspension instrument, the tail of the rats (about 1.5–2 cm) was fixed on the shelf, and the head was hung down about 35 cm away from the ground. A series of parameters during the process of the rats' desperate immobility were recorded within 360 s using the single blind method. The measurement index was immobility time during suspension. Eight rats were selected from each group, and the average value was recorded (22).

### Food Foraging Behavior Assessment

Food foraging behaviors include looking for food, storing and carrying food. According to the research of Yuduo, foraging behaviors involve advanced cognitive function, decision-making and investment ability for future plans. Prior to the experiment, a group of normal rats completed the foraging behavior experiment for 5 consecutive nights. The rats were deprived of food for 12 h on a 50 × 50 × 50 cm white open field before the experiment. From 7:00 A.M. to 7:00 P.M., the rats were placed separately in the open field, where there were 60 g cauliflower dolls in the small tray placed in the middle of a certain edge of the open field. The rats were allowed to freely use and carry the tray in the open field again, and there was no cage in the open field; the carrying time was from 7:00 P.M. to 7:00 A.M. The next day, the rats were weighed, followed by calculation of the consumption weight and transportation weight during one night (23).

### Immunohistochemistry

After anesthesia, the rats were euthanized immediately after cervical dislocation. The left ventricle was intubated approaching the aorta after thoracoscopy and quickly washed with 100 mL normal saline. The blood was collected quickly and fixed with 400 mL of 4% phosphate buffered saline (PBS) solution. Subsequently, the brain of rats was removed from the head (the brain tissues of 8 rats in each group were used for testing 5-hydroxytryptamine (5-HT), adrenocorticotropin (ACTH) and brain-derived neurotrophic factor (BDNF) content, and the rest were used for follow-up experiments). The brain tissues of rats in each group were cut into the same size of brain tissue slices containing the hippocampus using brain mold, and then fixed in 4% paraformaldehyde overnight. The right hippocampal tissues of rats in each group were sectioned in a continuous coronal position with a thickness of 10 μL. The BrdU and Nestin immunohistochemical staining assays were performed using the SABC method on the tissue sections of the same layer of rats in each group. When BrdU staining was carried out, 2N hydrochloric acid (37°C) was used to treat the tissues for 1 h to fully expose the antigen. The pathological tissue samples after color development were examined under a microscope, and 5 visual fields were randomly taken for taking photos, the results of which were analyzed by an image processing software. Positive brown granules in the cytoplasm in BrdU staining and those in the nucleus in Nestin staining indicated positive staining. The number of positive cells in each specimen was obtained from the average value of 5 visual fields.

### Hippocampal Neuron Isolation and Culture

Newborn Sprague-Dawley (SD) rats were provided by the Animal Center of Zhengzhou University. The hippocampal neurons were isolated from the newborn rats (24). First, the newborn SD rats were sacrificed under aseptic conditions. The skin and skull were cut, and the brain tissues were excised and placed in a cold pH 7.2, calcium and magnesium-free D-Hank's solution in a plate (with ice pack underneath). The cerebral cortex was carefully turned over the under a microscope with the back of the brain tissue facing up to expose the hippocampus, after which the tissues around the hippocampus were separated and placed in a dish filled with D-Hank's solution. The tissues were sectioned into about 1 mm^3^ blocks, treated with 0.125% trypsin, and incubated at 37°C for 20 min. Then Dulbecco's modified Eagle's medium (DMEM) (containing 10% FBS, 1% glutamine, 100 U/mL penicillin and 100 mg/L streptomycin) was added to terminate the digestion. Cell suspension was prepared by dispersing for 20 times with a pipette, which was allowed to stand for 2 min. The cell suspension was centrifuged (1,000 rpm, 10 min, 4°C) and the supernatant was harvested and resuspended in complete culture medium. The cells were filtered with 200 mesh cell sieve and sub-packaged into 96-well plates coated with 0.1 mg/mL polylysine. Next, 0.3 × 10^6^/mL cells were seeded into the 96-well plate in 9% CO_2_, 37°C incubator. After culturing for 36 h, 3 μg/mL cytarabine was added to inhibit the division and growth of glial cells, and the medium was changed after 12 h. Afterwards, half of the medium was changed every 3 days, and the experiment can be carried out after 7 to 14 days of *in vitro* culture. The hippocampal neurons were treated with corticosterone (CORT, dissolved in 0.1% dimethyl sulphoxide [DMSO]) for *in vitro* stimulation. In specifics, the cell culture medium was replaced with 100 μmol/L CORT serum-free DMEM (control group without any drugs) for 30 min of incubation, followed by infections and other operations.

### Lentiviral Infection

A lentiviral packaging system was constructed using LV5-green fluorescent protein (GFP) (lentiviral gene overexpression vector), and pSIH1-H1-copGFP (lentiviral gene silencing vector). oe-STC1 and the negative control oe-NC were constructed by Shanghai GenePharma Co., Ltd. (Shanghai, China). HEK293T cells were co-transduced with packaging virus and the target vector. The supernatant was collected after cell culture for 48 h. The supernatant contained virus particles after filtration and centrifugation, and the virus titer (about 10^12^ infection units/mL) was detected. The lentiviruses expressing oe-STC1, oe-NC, si-NC, and si-STC1 in the exponential phase were collected and grouped according to different infections. To block the ROS activity, cells were treated with NAC (ROS inhibitor; 100 μM; Selleck.cn, Shanghai, China) with DMSO (0.1%) as control. To block the NF-κB p65 signal, cells were treated with BAY117082 (NF-κB p65 inhibitor; 10 μM; Selleck.cn, Shanghai, China) with DMSO (0.1%) as the control. Before treatment, hippocampal neurons were detached with trypsin and pipetted to cell suspension of 5 × 10^4^ cells/mL, which was inoculated in a 6-well plate with 2 mL per well and cultured overnight at 37°C. NAC and BAY117082 were pretreated for 30 min before infection. After 48 h of infection, the mRNA and protein expression in the hippocampal neurons was measured by reverse transcription-quantitative polymerase chain reaction (RT-qPCR) and Western blot assay. The experiment was repeated 3 times.

### Cell Counting Kit-8 Assay

The CCK-8 assay (Beyotime, Shanghai, China) was utilized to measure the cell viability. The cells were seeded in a 96-well plate at a density of 8 × 10^3^ cells/well, and incubated in a constant temperature incubator for 24 h. Next, 10 μL of CCK-8 solution was added into the plate with 100 μL of medium per well. The absorbance of each well at a wavelength of 450 nm was recorded on a BioTek microplate reader (BioTek Instruments, Thermo Fisher Scientific, Winooski, VT, USA).

### Lactate Dehydrogenase Release Assessment

The cytotoxicity test kit PLUS (Roche, Germany) was utilized to determine the cellular release of LDH according to the manufacturer's protocol. Cytotoxicity was determined using a Varioskan microplate reader (Thermo Fisher Scientific) at 492 nm and a reference wavelength of 630 nm. The percentage of cytotoxicity is calculated by the following formula: Cytotoxicity = (OD_Sample_—OD_LowControl_)/(OD_MaximalRelease_—OD_LowControl_) × 100.

### Flow Cytometry

The cell apoptosis was assessed using Annexin V-fluorescein isothiocyanate (FITC)/propidium iodide (PI) double staining kit (Cat. No. 70-AP101-100, Hangzhou Duokexue (Lanke) Biotechnology Co., Ltd., Hangzhou, China). The cultured hippocampal neurons were detached with 0.25% trypsin without ethylenediamine tetraacetic acid (EDTA), and centrifuged at 300 × g for at least 5 min. After removing the supernatant, the remaining pellet was resuspended in 500 μL of binding buffer. Next, the cells were mixed with 3 μL Annexin V-FITC and 2.5 μL PI (Beyotime), and incubated in the dark at 37°C for 20 min. Then the cell apoptosis was analyzed by flow cytometer (FACS Calibur, BD Biosciences, San Jose, CA, USA). The experiment was repeated three times.

### RT-qPCR

The total RNA in the hippocampus was extracted by TRIzol one-step method based on the instructions of the TRIzol Kit (15596-026, Invitrogen, Gaithersburg, MD, USA). The optical density (OD) at 260 nm and 280 nm and RNA concentration were measured using a protein detector (BioPhotometer D30, Eppendorf, Hamburg, Germany). The OD_260nm_/OD_280nm_ ratio between 1.8 and 2.0 indicated high RNA purity. According to the instructions of the reverse transcription kit (K1621, Fermentas, Hanover, Maryland, USA), RNA was reverse-transcribed into complementary DNA (cDNA). The primer sequences for STC1, ROS, NF-κB p65, glutamate receptor 1 (GluR1), BDNF, glial fibrillary acidic protein (GFAP), S100β (Supplementary Table 1) were designed and synthesized by Genechem. The mRNA expression was determined using a fluorescence qPCR kit (Takara, Dalian, China) in a real-time fluorescent qPCR (ABI 7500, ABI, Foster City, CA, USA). Using glyceraldehyde-3-phosphate dehydrogenase (GAPDH) as internal reference, we applied the 2^−ΔΔCT^ method to calculate the relative expression of each target gene; this method was also used to determine the mRNA expression in cells.

### Western Blot Assay

The hippocampal tissues were lysed with the protein lysis buffer (R0010, Beijing Solarbio Science & Technology Co., Ltd., Beijing, China) at 3,000 r/minute until the homogenate was fully lysed. The protein concentration was determined according to the instructions of the bicinchoninic acid (BCA) protein quantitative kit (23225, Pierce, Rockford, IL, USA), and adjusted to 1 μg/μL. The treated protein was added to the sample well (20 μL per well), followed by electrophoresis separation with 10% sodium dodecyl sulfate-polyacrylamide gel electrophoresis gel (P1200, Solarbio). The protein samples were transferred to a polyvinylidene fluoride membrane (HVLP04700, Millipore, Bedford, MA, USA) by semi-dry transfer method, and then stained with fuchsin (P0012, Solarbio) to observe the protein transfer. After being blocked with 5% skimmed milk powder at room temperature for 2 h, the samples were probed with primary rabbit antibodies against STC1 (1: 1000, ab229477), ROS (1: 1000-1: 10000, ab181113), NF-κB p65 (1: 10000, ab16502), GluR1 (1: 2000, ab109450), BDNF (1: 100, ab108319), GFAP (1: 10000, ab7260), S100β (1: 1000, ab52642) and β-actin (1: 1000, ab8227), followed by overnight incubation in a 4°C refrigerator. The following day, the samples were incubated at room temperature for 2 h with the secondary antibody goat anti-rabbit against immunoglobulin (IgG; 1: 2000, ab6721), followed by diaminobenzidine color development. A gel imaging device Gel Doc XR (Bio-Rad Laboratories, Hercules, CA, USA) was applied for photographing. All the antibodies were purchased from Abcam (Cambridge, UK). The ratio of the gray value of the target protein to that of internal reference was regarded as the relative protein expression. This method was also used to determine the protein expression in cells.

### Enzyme-Linked Immunosorbent Assay

The hippocampal tissues of rats were added with 2 mL PBS (pH 7.4), and made into homogenate in a tissue homogenizer. The homogenate was centrifuged in a centrifuge tube at 4°C at 3,000 rpm for 20 min, the supernatant of which was restored in a 1.5 mL centrifuge tube at −80°C. The levels of 5-HT, ACTH and BDNF in the whole brain were determined following the instructions of the ELISA 5-HT (ml003125), ACTH (ml002875), and BDNF (ml302829) kits from mlbio Co., Ltd. (Shanghai, China).

The levels of interleukin (IL)-1β, IL-6, tumor necrosis factor-α (TNF-α), high sensitivity C-reactive protein (hs-CRP) and interferon γ (IFN-7) were also detected by ELISA kits (IL-1β, ml037361; IL-6, ml064292; TNF-?, ml002859; hs-CRP, ml003217; IFN-7, ml064291) from mlbio Co., Ltd. The antigen was diluted to 1–10 μg/mL with carbonate coated buffer (pH 9.6), and added to reaction wells (0.1 mL for each well), followed by resting overnight at 4°C. The next day, 0.1 mL of diluted supernatant was added to the coated reaction well, and incubated at 37°C for 1 h; meanwhile, the blank, NC and positive control wells were set up, which were added with 0.1 mL of fresh diluted enzyme-labeled secondary antibodies (Abcam), followed by incubation for 35–60 min at 37°C. At last, the samples were rinsed with ddH_2_O (PER 018-1, Beijing Dingguo Changsheng Biotechnology Co., Ltd., Beijing, China). Finally, 50 μL of termination solution was used to stop color development, followed by measurement of the OD value in each well at the wavelength of 450 nm within 20 min.

### Terminal Deoxynucleotidyl Transferase-Mediated dUTP-Biotin Nick End Labeling (TUNEL) Staining

The hippocampal tissues of rats were fixed with 10% neutral formaldehyde solution for 24 h, and conventionally dehydrated with gradient alcohol (70, 80, 90, 95, and 100%), each for 1 min. The tissues were cleared with xylene 2 times, 5 min for each, soaked, embedded in paraffin, and sectioned into 4-μm-thick sections. The paraffin-embedded sections were dewaxed, immersed in 0.3% H_2_O_2_ solution, and allowed to stand at room temperature for 30 min. The apoptotic cells in the tissue section were detected using TUNEL kit (Roche Diagnostics GmbH, Mannheim, Germany). The specific steps were as follows: the sections were dewaxed with xylene, then hydrated with gradient alcohol, rinsed twice with PBS, each time for 3 min, and detached with protease K (20 μg/mL, 10 mM tris/HCl, pH = 7.5–8.0) in a 37°C incubator for 30 min. Each section was incubated with 25 μL of TUNEL reaction solution for 60 min and then with 25 μL of alkaline phosphatase antibody for 30 min. Next, the sections were counterstained with nuclear fast red, cleared, directly sealed with water-based sealer, and dried at 60°C. Afterwards, 10 fields of vision (no <100 cells) were randomly chosen under a light microscope, to count the number of positive cells: apoptotic rate = the number of apoptotic cells / total cells × 100%, and the average value was calculated.

### Determination of the Activity of Superoxide Dismutase (SOD) and Catalase (CAT) and the Level of Malondialdehyde (MDA) in the Hippocampus

A total of 10 mg hippocampal tissues were collected from normal rats and rats with depression-like behaviors, washed with 4°C normal saline, dried with filter paper and made into homogenate with homogenizer on ice bath. The homogenate was then centrifuged at 4,000 r/minute for 15 min, after which supernatant was subpacked into Eppendorf tube, and stored at −20°C. In addition, cell supernatant was harvested from the hippocampal neurons. Based on the instructions provided by the kits (mlbio, Shanghai, China), the activity of SOD (ml077379) and the levels of CAT (ml037752) and MDA (ml077384) in hippocampal tissues and cell supernatant were detected. The activity of SOD was determined *via* the xanthine oxidase method, and MDA level was measured with the absorbance of the red product produced by the reaction between SOD and thiobarbital sodium.

### Reactive Oxygen Species Activity Detection

The ROS activity in tissues was detected using tissue ROS detection kit (DHE, Biolab, Beijing, China). The tissues (50 mg) were washed with PBS and added with 1 mL of homogenization buffer A, and homogenized thoroughly with a glass homogenizer. After centrifugation at 1,000 g for 10 min at 4°C, the precipitate was removed, and the supernatant was collected. Next, 190 μL of homogenate supernatant and 10 μL of DHE probe were loaded onto a 96-well plate, and mixed thoroughly. Incubation was performed for 30 min at 37°C in the dark. The fluorescence intensity was detected in a fluorescence microplate reader, at an excitation wavelength of 488–535 nm and an emission wavelength of 610 nm. Additionally, 50 μL homogenate supernatant was diluted with PBS ~30 times and then 100 μL of which was used for protein quantification. Fluorescence intensity/mg protein represents the intensity of ROS production in tissue.

A ROS detection kit (Beyotime, Shanghai, China) was used to determine the accumulation of ROS production in cells, with DCFH-DA as a fluorescent probe. The cells were incubated with 10 mm DCFH-DA at 37 C for 30 min. The intracellular ROS production was detected on a fluorescence microscope at excitation wavelength of 488 nm and emission wavelength of 525 nm.

### Statistical Analysis

The data were analyzed by SPSS 21.0 (IBM Corp. Armonk, NY, USA). The measurement data were expressed as mean ± standard deviation. One-way analysis of variance (ANOVA) with Tukey's *post hoc* test was utilized for multi-group data comparison. Repeated measures of ANOVA with Bonferroni *post hoc* test was used to compare data at different time points. *p* < 0.05 demonstrated statistically significant difference.

## Results

### Successful Establishment of a Rat Model With Depression-Like Behaviors

**Figure 1:**
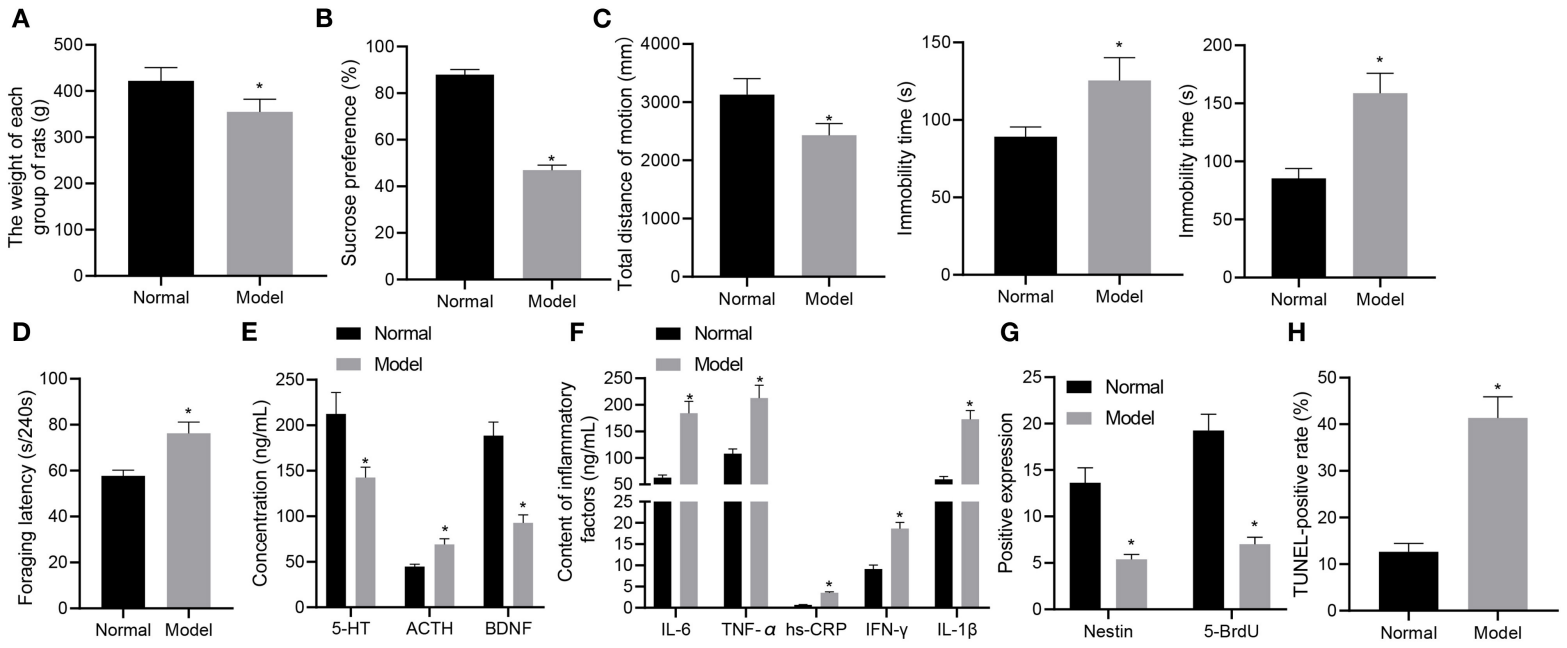
A rat model of depression-like behaviors is successfully established. **(A)** weight of rats with depression-like behaviors. **(B)** sucrose preference in rats with depression-like behaviors. **(C)** total movement distance and cumulative immobility time of rats with depression-like behaviors in the forced-swimming test and tail suspension experiment. **(D)** latency time of food foraging of rats with depression-like behaviors. **(E)** 5-HT, BDNF, and ACTH levels in the serum of rats with depression-like behaviors. **(F)** IL-1β, IL-6, TNF-α, hs-CRP, and IFN-γ levels in serum of rats with depression-like behaviors. **(G)** numbers of BrdU- and Nestin-positive cells in the hippocampus of rats with depression-like behaviors. **(H)** The percentage of TUNEL-positive cells in the hippocampus of rats with depression-like behaviors. *p < 0.05. *n* = 8.

Initially, we developed a rat model of depression-like behaviors using chronic unpredictable mild stress (CUMS) method and measured the weight of rats with different treatments. At the 8th week, the mean weight of normal rats was (422.03 ± 28.67) g, compared to which the weight of rats experiencing stress stimulation (354.67 ± 27.58) g was significantly reduced (*p* < 0.05) (Figure 1A). Subsequently, the results of sucrose preference test showed that the average sucrose preference in the normal rats was (87.96 ± 2.18)%, which was reduced in stress-stimulated rats (46.99 ± 2.17)% (*p* < 0.05) (Figure 1B). As depicted in Figure 1C, open-field test exhibited that the total movement distance was significantly shortened in the rats experiencing stress stimulation as compared to normal rats (*p* < 0.05). In the forced-swimming and tail suspension tests, as shown in Figure 1C, the cumulative immobility time was significantly increased in the rats experiencing stress stimulation. In the food foraging experiment, the latency time of food foraging in normal rats was (57.69 ± 2.49) seconds/240 s, which was reduced in the rats experiencing stress stimulation (Figure 1D). These data suggested that the rats had locomotor deficit and developed depression-like behaviors after stress stimulation.

ELISA was performed to determine the expression of depression-related and inflammatory factors in rats with different treatments. As shown in Figures 1E,F, the levels of 5-HT and BDNF were appreciably lowered and those of ACTH, IL-1β, IL-6, TNF-α, hs-CRP, and IFN-γ were notably lower in stress-stimulated rats than in normal rats (*p* < 0.05). Immunohistochemistry results (Figure 1G) showed that BrdU and Nestin were strongly positive in the hippocampus of the normal rats, while BrdU- and Nestin-positive cells were reduced in the hippocampus of rats experiencing stress stimulation (*p* < 0.05). TUNEL staining further showed that the percentage of TUNEL-positive cells in the hippocampus of normal rats was lower than that of rats experiencing stress stimulation (*p* < 0.05) (Figure 1H). These results demonstrated the successful establishment of the rat model with depression-like behaviors.

### STC1 Is Poorly Expressed While ROS/NF-κB Signaling Pathway Is Activated in Rats With Depression-Like Behaviors

**Figure 2:**
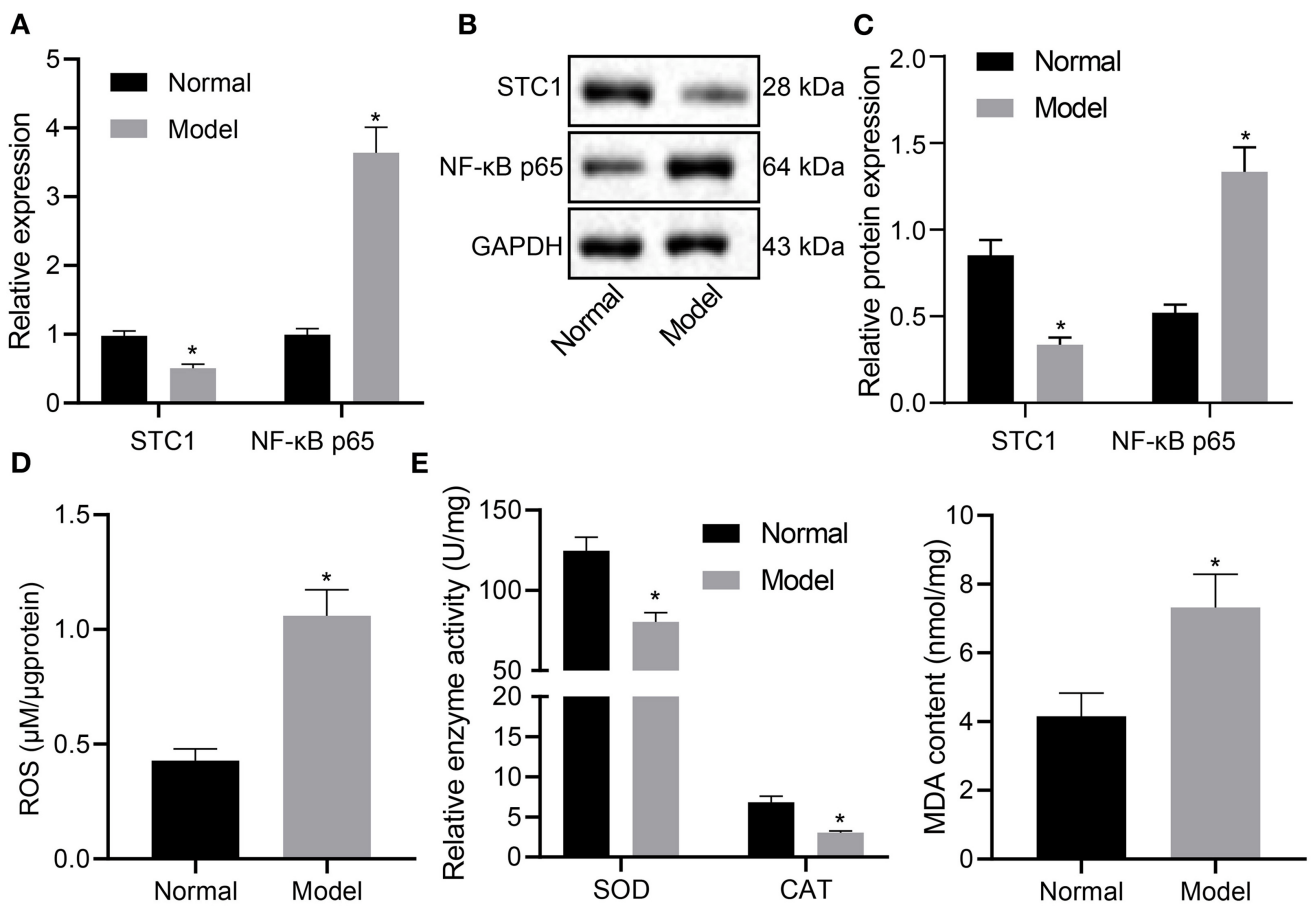
STC1 is underexpressed while ROS/NF-κB signaling pathway is activated in enhances rats with depression-like behaviors. **(A)** STC1 and NF-κB p65 mRNA expression in the hippocampal tissues of rats with depression-like behaviors determined by RT-qPCR. **(B)** STC1 and NF-κB p65 protein expression in the hippocampal tissues of rats with depression-like behaviors measured by Western blot assay. **(C)** Quantitative analysis of STC1 and NF-κB p65 protein expression. **(D)** ROS production in the hippocampal tissues of rats with depression-like behaviors. **(E)** SOD, CAT, and MDA levels in hippocampal tissues of rats with depression-like behaviors. *p < 0.05. *n* = 8.

STC1, a 56 kDa glycoprotein hormone, shows neuroprotective role in hypercalcemic and hypoxic damage in brain neurons (10). Next, we experimentally characterized the mRNA and protein expression patterns of STC1, and ROS/NF-κB signaling pathway-related genes in the hippocampus of rats with different treatments. The results of RT-qPCR and Western blot assays showed that the mRNA and protein expression of STC1 in the hippocampus of stress-stimulated rats was significantly lower than that of normal rats, and that of NF-κB p65 was notably higher (*p* < 0.05) (Figures 2A–C).

Meanwhile, the ROS production was determined to be significantly increased in stress-stimulated rats (*p* < 0.05) (Figure 2D). The levels of oxidative stress markers were also detected. Relative to that in the normal rats, SOD activity and CAT activity in rats experiencing stress stimulation decreased significantly, accompanied with notably increased MDA level (*p* < 0.05) (Figure 2E). Taken together, STC1 was under-expressed and the ROS/NF-κB signaling pathway was activated in the rat model of depression-like behaviors.

### STC1 Suppress the Toxicity and Apoptosis of Hippocampal Neurons Induced by CORT

**Figure 3:**
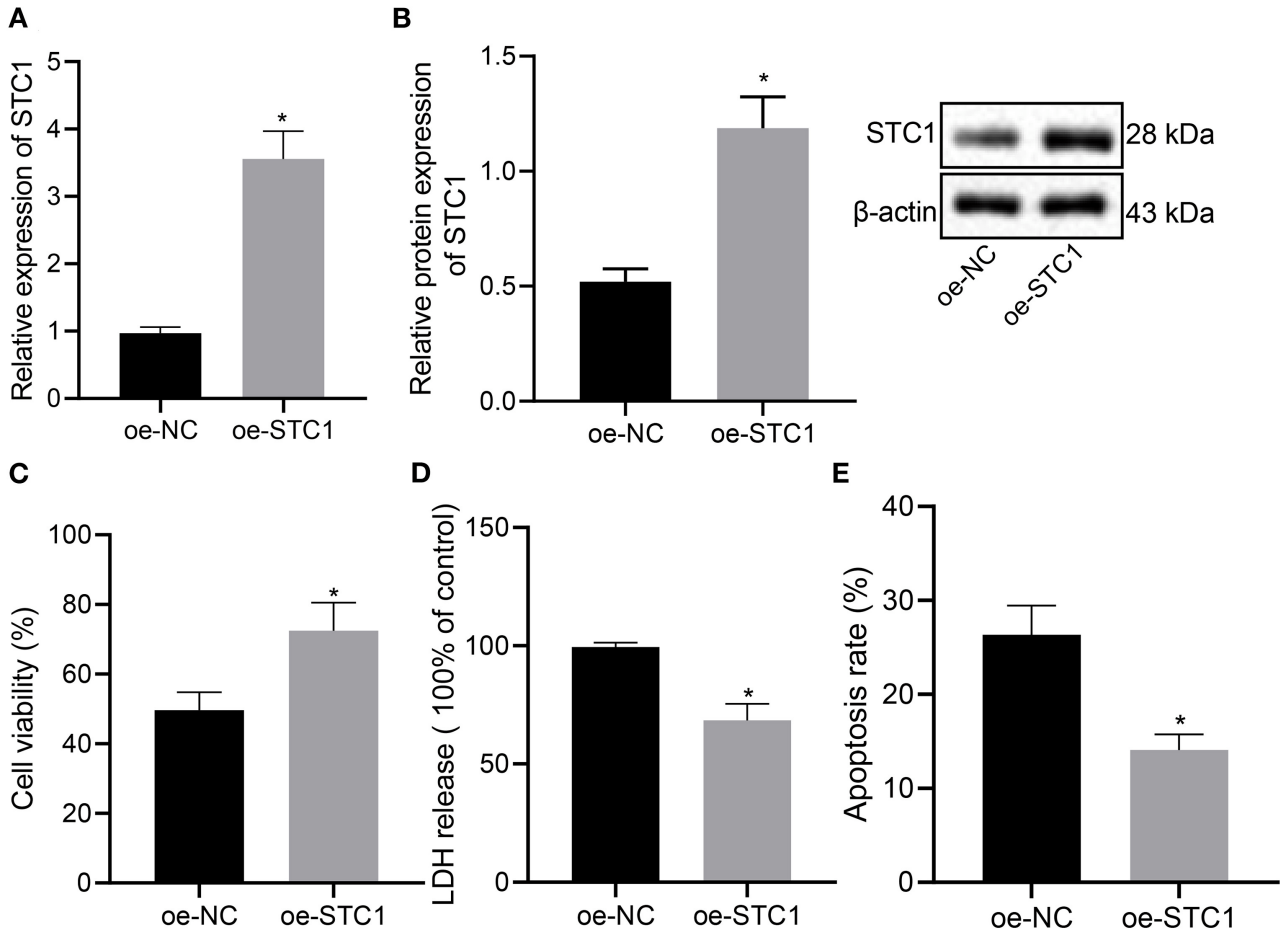
STC1 overexpression reduces the toxicity and apoptosis of hippocampal neurons induced by CORT. **(A)** STC1 mRNA expression in hippocampal neurons induced by CORT determined by RT-qPCR. **(B)** STC1 protein expression in hippocampal neurons induced by CORT measured by Western blot assay. **(C)** viability of hippocampal neurons induced by CORT. **(D)** LDH activity in hippocampal neurons induced by CORT. **(E)** apoptosis of hippocampal neurons induced by CORT. *p < 0.05 *vs*. neurons treated with oe-NC. Cell experiment was repeated 3 times.

We further explored the effect of overexpression of STC1 on the viability, toxicity and apoptosis of hippocampal neurons by culturing primary hippocampal neurons *in vitro*. The results of RT-qPCR and Western blot assay showed that compared with the oe-NC-treated neurons, the mRNA and protein expression of STC1 increased significantly in the hippocampal neurons treated with oe-STC1 (Figures 3A,B). Additionally, the results of CCK-8 assay presented that oe-STC1 enhanced the viability of hippocampal neurons (Figure 3C). As revealed by LDH activity test and flow cytometric data, the toxicity and apoptosis of hippocampal neurons were significantly reduced by oe-STC1 (Figures 3D,E). The above-mentioned results indicated that overexpression of STC1 could reduce neuronal toxicity and apoptosis caused by CORT.

### STC1 Inhibits Activation of the ROS/NF-κB Signaling Pathway

**Figure 4:**
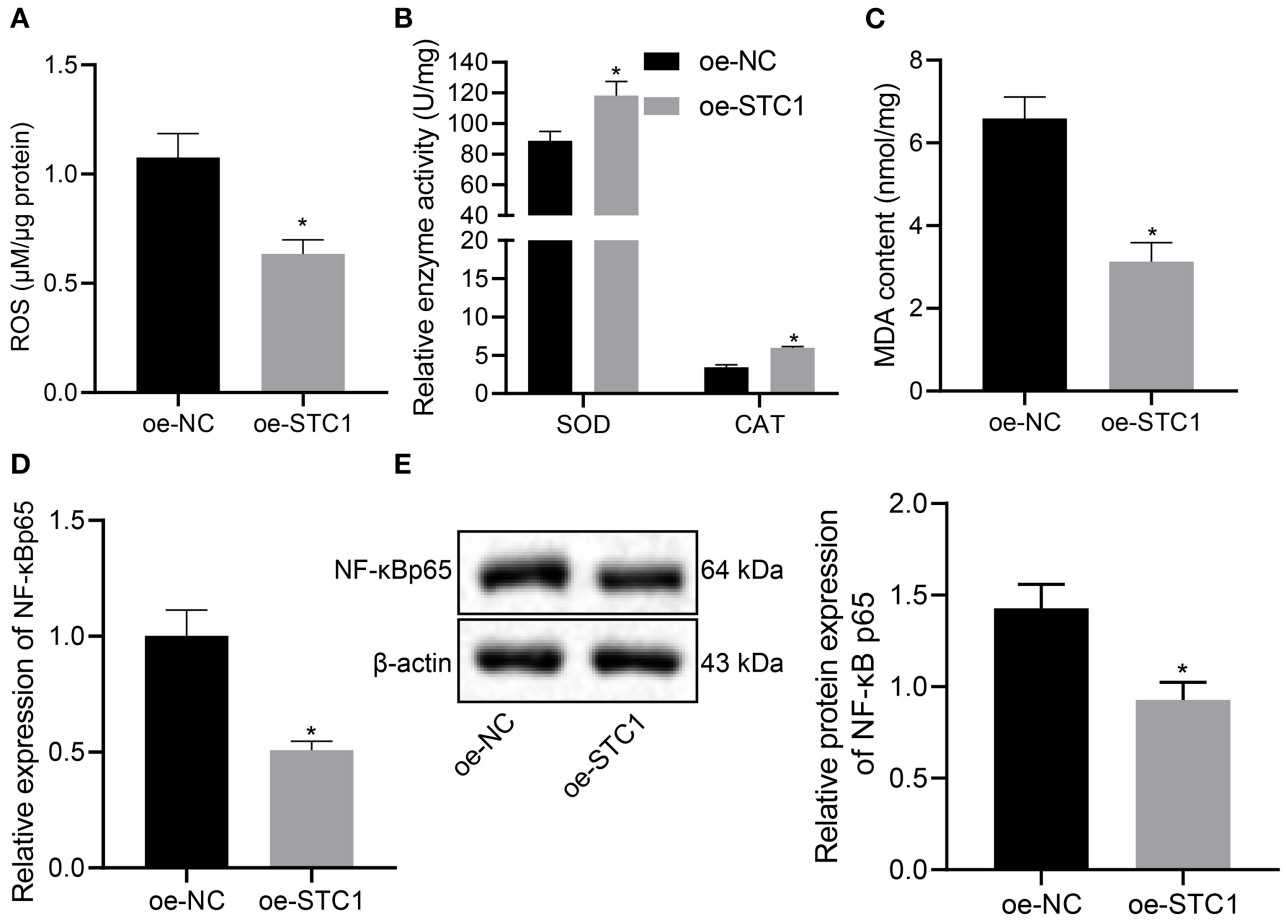
STC1 overexpression inhibits the ROS/NF-κB signaling pathway. **(A)** ROS production in hippocampal neurons induced by CORT. **(B)** SOD and CAT activity in hippocampal neurons induced by CORT. **(C)** MDA level in hippocampal neurons induced by CORT. **(D)** NF-κB p65 mRNA expression in hippocampal neurons induced by CORT determined by RT-qPCR. **(E)** NF-κB p65 protein expression in hippocampal neurons induced by CORT measured by Western blot assay. *p < 0.05 *vs*. neurons treated with oe-NC. Cell experiment was repeated 3 times.

Next, we overexpressed STC1 in hippocampal neurons to explore its effect on ROS/NF-NF-κB signaling pathway and oxidative stress markers. As depicted in Figure 4A, oe-STC1 treatment led to a reduction in ROS production in hippocampal neurons. Additionally, SOD activity and CAT activity in neurons were elevated significantly, accompanied with notably diminished MDA level after oe-STC1 treatment (Figures 4B,C). Compared with the oe-NC-treated neurons, the mRNA and protein expression of NF-κB p65 in the neurons treated with oe-STC1 displayed a marked decline (*p* < 0.05) (Figures 4D,E). Thus, STC1 overexpression blocked the ROS/NF-κB signaling pathway.

### STC1 Prevents CORT-Induced Toxicity and Apoptosis of Hippocampal Neurons Through Blocking the ROS/NF-κB Signaling Pathway

**Figure 5:**
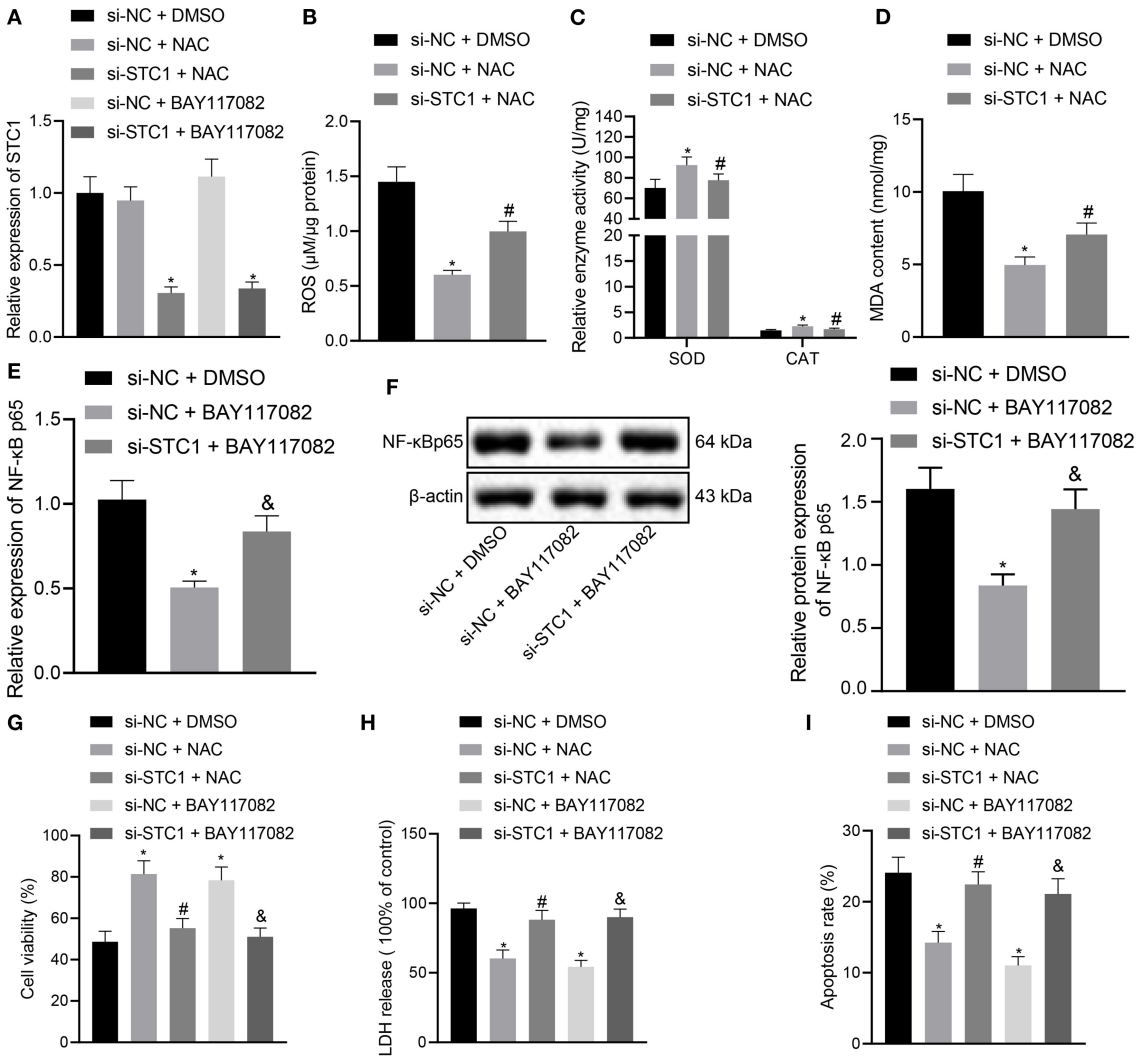
STC1 overexpression inhibits the ROS/NF-κB signaling pathway to repress toxicity and apoptosis of hippocampal neurons induced by CORT. **(A)** STC1 mRNA expression in hippocampal neurons induced by CORT determined by RT-qPCR after different treatments. **(B)** ROS production in hippocampal neurons induced by CORT after different treatments. **(C)** SOD and CAT activity in hippocampal neurons induced by CORT after different treatments. **(D)** MDA level in hippocampal neurons induced by CORT after different treatments. **(E)** NF-κB p65 mRNA expression in hippocampal neurons induced by CORT determined by RT-qPCR after different treatments. **(F)** NF-κB p65 protein expression in hippocampal neurons induced by CORT measured by Western blot assay after different treatments. **(G)** viability of hippocampal neurons induced by CORT after different treatments. **(H)** LDH activity in hippocampal neurons induced by CORT after different treatments. **(I)** apoptosis of hippocampal neurons induced by CORT after different treatments. *p < 0.05 *vs*. neurons treated with si-NC + DMSO. ^#^*p* < 0.05 *vs*. neurons treated with si-NC + NAC. ^&^*p* < 0.05 *vs*. neurons treated with si-NC + BAY117082. Cell experiment was repeated 3 times.

The *in vitro* experiments were performed to further explore the effect of STC1-mediated ROS/NF-κB signaling pathway on the viability, toxicity and apoptosis of hippocampal neurons using ROS and NF-κB inhibitors. The results of RT-qPCR showed that the mRNA and protein expression of STC1 was reduced in the hippocampal neurons treated with si-STC1 and NAC or si-STC1 and BAY117082 as compared to neurons treated with si-NC and NAC or si-NC and BAY117082, respectively (Figure 5A). NAC treatment appreciably diminished ROS and MDA levels and notably elevated SOD activity and CAT activity in the neurons treated with si-NC, while STC1 knockdown reversed these effects of NAC treatment (Figures 5B–D). BAY117082 treatment reduced the mRNA and protein expression of NF-κB p65 in the neurons treated with si-NC, which was rescued after STC1 knockdown (Figures 5E,F). Either NAC or BAY117082 treatment elevated the viability of hippocampal neurons and reduced the LDH activity (Figures 5G,H). The apoptosis of hippocampal neurons was significantly reduced by NAC or BAY117082 treatment (Figure 5I). However, STC1 knockdown reversed the effects of NAC or BAY117082 treatment on neuron viability, toxicity and apoptosis. The above-mentioned results indicated that STC1 silencing could induce neuronal toxicity and apoptosis caused by CORT through activating the ROS/NF-κB signaling pathway.

### STC1 Inhibits Inflammation and Relieves Depression-Like Symptoms Through Blocking the ROS/NF-κB Signaling Pathway

**Figure 6:**
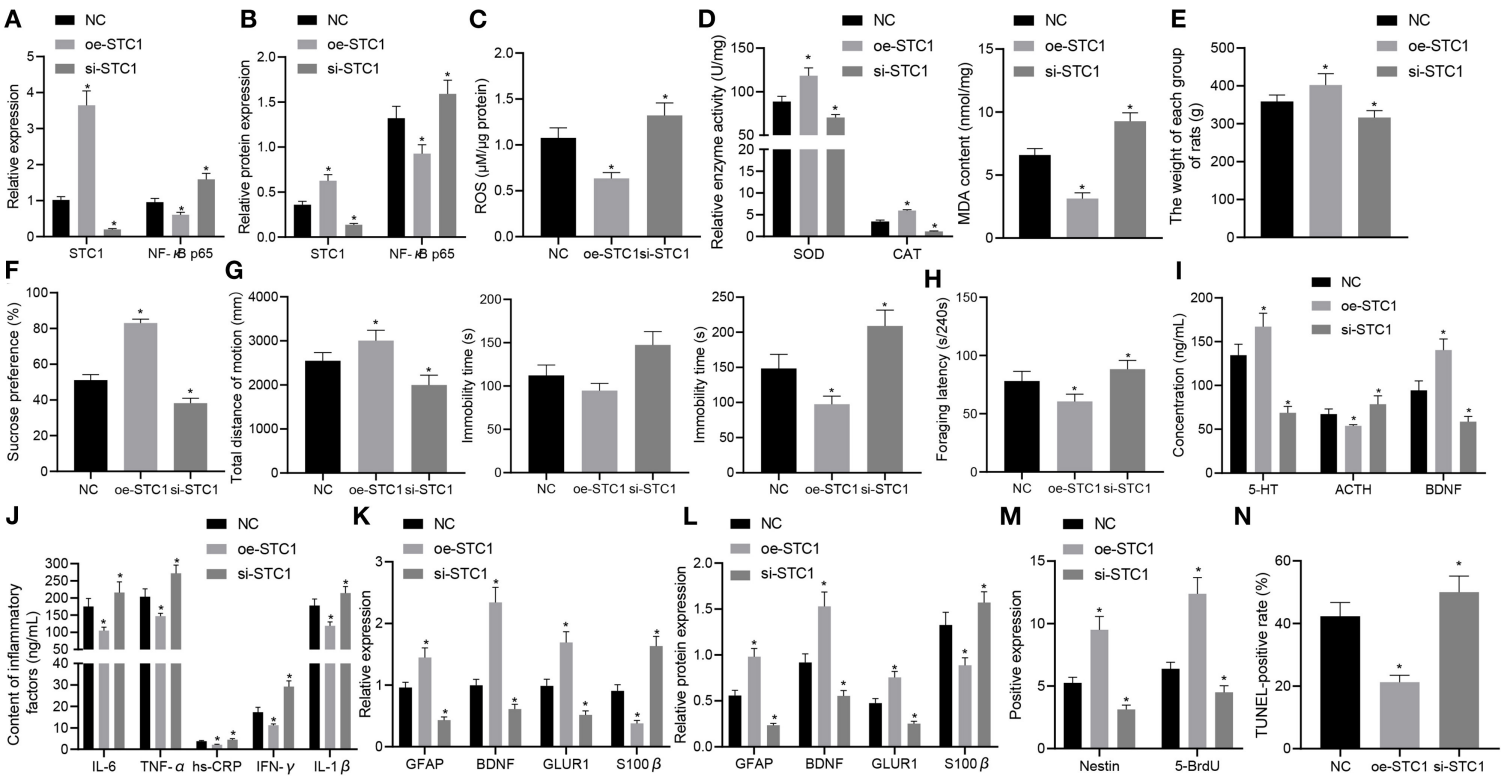
STC1 overexpression reduces depression-like behaviors. **(A)** STC1 and NF-κB p65 mRNA expression in the hippocampal tissues of rats with depression-like behaviors determined by RT-qPCR. **(B)** STC1 and NF-κB p65 protein expression in the hippocampal tissues of rats with depression-like behaviors measured by Western blot assay. **(C)** ROS production in the hippocampal tissues of rats with depression-like behaviors. **(D)** SOD, CAT, and MDA levels in the hippocampal tissues of rats with depression-like behaviors. **(E)** weight of rats with depression-like behaviors. **(F)** sucrose preference in rats with depression-like behaviors. **(G)** total movement distance and cumulative immobility time of rats with depression-like behaviors in the forced-swimming test and tail suspension experiment. **(H)** latency time of food foraging of rats with depression-like behaviors. **(I)** 5-HT, BDNF, and ACTH levels in the serum of rats with depression-like behaviors. **(J)** IL-1β, IL-6, TNF-α, hs-CRP, and IFN-γ levels in serum of rats with depression-like behaviors. **(K)** expression of neural plasticity-related genes (BDNF, GIUR1 and GFAP) and S100β determined by RT-qPCR. **(L)** expression of neural plasticity-related proteins (BDNF, GIUR1 and GFAP) and S100β measured by Western blot assay. **(M)** numbers of BrdU- and Nestin-positive cells in the hippocampus of rats with depression-like behaviors. **(N)** percentage of TUNEL-positive cells in the hippocampus of rats with depression-like behaviors. *p < 0.05 *vs*. stress-stimulated rats treated with oe-NC. *n* = 8.

Finally, the effects of STC1-mediated ROS/NF-κB signaling pathway on the depression-like symptoms *in vivo*. The STC1 expression *in vivo* was successfully enhanced or silenced using AAV, and STC1 overexpression reduced mRNA and protein expression of NF-κB p65 while STC1 silencing elevated that of NF-κB p65 (Figures 6A,B). Consequently, STC1 overexpression led to a reduction in ROS production in the hippocampal tissues, which could be elevated by STC1 silencing (Figure 6C). SOD activity and CAT activity in hippocampal tissues were elevated significantly, and MDA level was notably diminished after STC1 overexpression, all of which were reversed after STC1 silencing (Figure 6D).

The weight of stress-stimulated rats was significantly increased after STC1 overexpression, but notably decreased by STC1 silencing (Figure 6E). In addition, STC1 overexpression resulted in enhanced sucrose preference (Figure 6F), increased movement distance (Figure 6G), reduced immobility time (Figure 6G), and increased latency time of food foraging (60.57 ± 6.21) s/240 s (Figure 6H) in the stress-stimulated rats. However, the stress-stimulated rats treated with si-STC1 exhibited diminished sucrose preference, reduced movement distance, increased immobility time, reduced latency time of food foraging (88.27 ± 7.57) s/240 s (*p* < 0.05). Furthermore, STC1 overexpression appreciably elevated the levels of 5-HT and BDNF but reduced those of ACTH, IL-1β, IL-6, TNF-α, hs-CRP, and IFN-γ in stress-stimulated rats, while STC1 silencing led to a marked decrease in the levels of 5-HT and BDNF and an increase in those of ACTH, IL-1β, IL-6, TNF-α, hs-CRP, and IFN-γ (Figures 6I,J).

We also examined the levels of neural plasticity-related genes (BDNF, GIUR1 and GFAP) and S100β after intervention. The results suggested that the mRNA and protein expression of BDNF, GIUR1, and GFAP was significantly increased in the stress-stimulated rats in response to STC1 overexpression, and that of S100β displayed a marked decline, all of which were opposite to these changes induced by STC1 silencing (Figures 6K,L). Meanwhile, re-expression of STC1 significantly increased the number of BrdU- and Nestin-positive cells (Figure 6M) and percentage of TUNEL-positive cells (Figure 6N) in the hippocampus of stress-stimulated rats. By contrast, STC1 knockdown reduced the number of BrdU- and Nestin-positive cells and percentage of TUNEL-positive cells. The aforementioned data demonstrated that STC1 overexpression could suppress the inflammation and apoptosis in the hippocampus as well as relieving the symptoms of rats with depression-like behaviors.

## Discussion

In this study, we mainly illuminated that STC1 was not only capable of protecting stress stimulation-induced depression-like behaviors but also able to repress the release of pro-inflammatory proteins and enhance the neural plasticity in rats with depression-like behaviors *via* mediation of the ROS/NF-κB signaling pathway.

A downregulated expression of STC1 was observed in the rats after stress stimulation. According to the results of behavior tests including sucrose preference, open-field, forced-swimming, tail suspension and the foraging tests, we found that STC1 considerably reduced the depression-like behaviors induced by stress stimulation in rats. Intriguingly, a previous study discovered that STC1 concentration was decreased in patients with dementia relative to those Alzheimer's disease and cognitively normal controls (25). Similar to our findings, mammalian STC1 could exert neuroprotective effect against hypercalcemic and hypoxic injury in brain neurons (10). Our study demonstrated that STC1 elevated the numbers of BrdU- and Nestin-positive cells in rats with depression-like behaviors, suggestive of its beneficial role in neurogenesis. In addition to these effects, STC1 exert suppressive effect on inflammation and promotive effect on neural plasticity in the rats with depression-like behaviors. Also, STC1 was found to increase the activity of SOD and CAT in the hippocampus of a cerebral ischemia/reperfusion rat model, thereby reducing oxidative stress and blood brain barrier permeability to ameliorate cerebral ischemia (26), which was consistent with our findings. Furthermore, STC1 was identified as an ROS scavenger in the research conducted by Liu et al. They found that STC1 could improve anti-inflammation, anti-oxidant as well as anti-apoptosis activities by increasing SOD and decreasing MDA, IL-6, and IFN-γ through downregulation of ROS production (12). Besides, inhibition of ROS overproduction and NF-κB activation diminished the levels of chemokines in endothelial cells, which was found to be in part attributed to STC1-upregulated uncoupling protein 2 (27). Hence, we further analyzed whether STC1 could mediate the ROS/NF-κB signaling pathway in rats with depression-like behaviors.

The ROS/NF-κB signaling pathway has been extensively reported to regulate inflammation and neural plasticity. For instance, resveratrol-activated ROS/NF-κB signaling pathway aggravated phosphine-induced hepatic injury partially by reducing SOD and CAT, increasing the levels of TNF-α, IL-1β, and IL-6, and promoting apoptosis of hepatocytes (28). The aerobic exercise-induced downregulation of the NF-κB/NLRP3/IL-1β signaling pathway could increase the expression of proteins related to hippocampal synaptic plasticity in diabetic rats (29). Moreover, inhibition of NF-κB by hesperidin could aid in diminishing neuronal degeneration and hippocampal inflammation, while improving learning as well as cognitive responses in a sevoflurane anesthetized neonatal rat model (30). In addition, the activated NF-κB pathway could contribute to enhancing the associative plasticity in an aged neural network and is therefore potential for treating age-related cognitive decline (31).

Besides, ROS could mediate intermittent hypoxia to reduce adult neurogenesis as well as synaptic plasticity in the dentate gyrus in the hippocampus, and the resultant neurophysiological changes may lead to deficits in cognition and behavior occurring in sleep apnea (32). In addition, several researches have reported the involvement of the ROS/NF-κB signaling pathway in the development of neural diseases. It is thought that NF-κB shares correlation with inflammation and ROS production in various disorders including depression (33). Consistent with our results, inhibited NF-κB/NLRP3 inflammasome pathway was found to ameliorate depressive-like behaviors in rats with chronic unpredictable mild stress (34). Similarly, Wu et al. found that ROS production was increased in the hippocampus of aging mice with isoflurane-induced cognitive deficits (35). ROS production in human brain cells participated in the neuropathologic mechanisms in Alzheimer's disease and Parkinson's disease (36). In addition, upregulation of STC1 was reported to suppress ROS production in mice with renal ischemia/reperfusion injury (37). More importantly, STC1 secreted by mesenchymal stem cells could play a neuroprotective role in acute glaucoma partially by downregulating the levels of inflammatory factors such as TNF-α, IL-1β, and ROS production, while inhibiting the apoptosis of retinal ganglion cells (38). Our *in vitro* experiments demonstrated that STC1 silencing could activate the ROS/NF-κB signaling pathway to induce the toxicity and apoptosis of neurons induced by CORT. Our findings suggested that the anti-inflammatory role of STC1 and its neuroprotective in depression-like behaviors might be achieved by mediation on the ROS/NF-κB signaling pathway. However, there are still some limitations of the present study. On the one hand, the immobility in the forced swim has been suggested to be related more to stress coping and adaptation. We may adopt other approaches to establish and evaluate animal models with depression-like behaviors in future experiments. On the other hand, attention should be paid to the physiological and patho-physiological differences when relating the animal results to the human clinical setting, and further experiments on humans are thus required.

## Conclusions

To sum up, the current study demonstrated an alleviatory role of STC1 in depression-like behaviors by suppressing the release of inflammatory factors and enhancing neuron functions, which is achieved by inhibiting the ROS/NF-κB signaling pathway. This finding may provide a novel neuroprotective target for treatment of depression. Nevertheless, the specific mechanisms regarding the role of ROS and NF-κB in depression still need further exploration.

## Data Availability Statement

The original contributions presented in the study are included in the article/Supplementary Material, further inquiries can be directed to the corresponding author/s.

## Ethics Statement

The animal study was reviewed and approved by Guang'anmen Hospital, China Academy of Chinese Medicine.

## Author Contributions

BC, LZ, and SH: conceptualization. JP, YZ, and YC: data curation and formal analysis. BC and MX: investigation and methodology. SH: project administration and supervision. BC: resources. LZ and YZ: software. LZ: validation. BC, LZ, and JP: writing—original draft. YZ, YC, MX, and SH: writing—review & editing. All authors contributed to the article and approved the submitted version.

## Conflict of Interest

The authors declare that the research was conducted in the absence of any commercial or financial relationships that could be construed as a potential conflict of interest.

## Supplementary Material

Supplementary Figure 1 A schematic diagram of the timeline of the experiments.

Supplementary Table 1 Primer sequences for RT-qPCR.
