# Supplementary figures and images for: Stanniocalcin-1 Overexpression Prevents Depression-Like Behaviors Through Inhibition of the ROS/NF-κB Signaling Pathway
Source: Front Psychiatry. 2021 Jun 14;12:644383. doi: 10.3389/fpsyt.2021.644383 (PMC8238083; doi:10.3389/fpsyt.2021.644383)

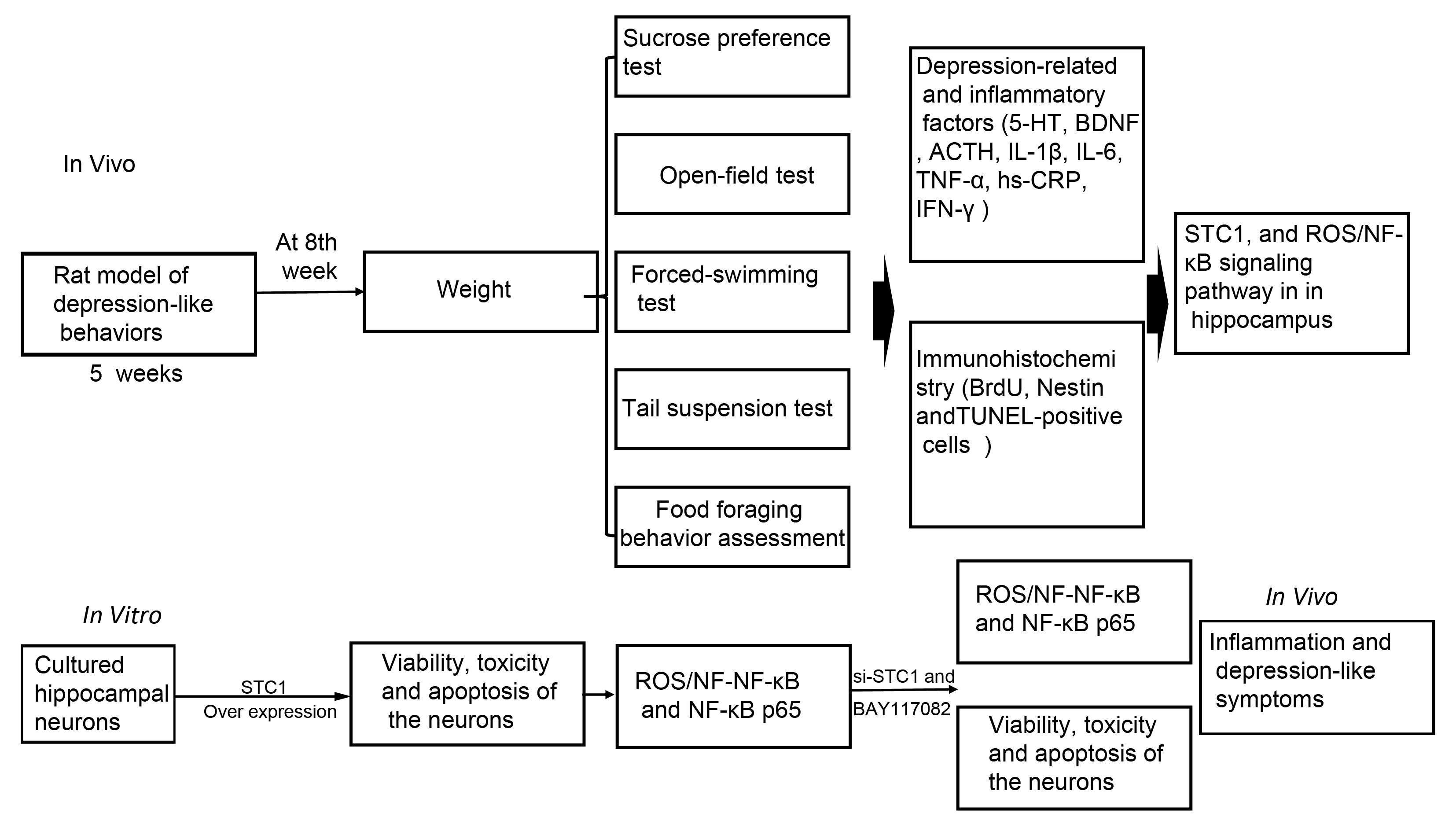

Supplement: Supplementary Figure 1 — A schematic diagram of the timeline of the experiments. [file Image_1.JPEG]
