# Supplementary material for: Stanniocalcin-1 Overexpression Prevents Depression-Like Behaviors Through Inhibition of the ROS/NF-κB Signaling Pathway
Source: Front Psychiatry. 2021 Jun 14;12:644383. doi: 10.3389/fpsyt.2021.644383 (PMC8238083; doi:10.3389/fpsyt.2021.644383)
Supplement: Supplementary Table 1 — Primer sequences for RT-qPCR. [file Table_1.DOCX]

**TABLE S1 |** Primer sequences for RT-qPCR

| Gene | Sequences |
| --- | --- |
| STC1 | F: 5′-CTCACGAGGCGGAACAGAAT-3' |
|  | R: 5′-CCGAATGGCAAGGAAGACCT-3' |
| NF-κB p65 | F: 5′-CATGGATCCCTGCACACCTT-3' |
|  | R: 5′-CTCAGCATGGAGAGTTGGCA-3' |
| GluR1 | F: 5′-GGACAACTCAAGCGTCCAGA-3'  R: 5′-CACAGTAGCCCTCATAGCGG-3' |
| BDNF | F: 5′-CCAATCGAAGCTCAACCGAA-3' |
|  | R: 5′-GGGAACCCGGTCTCATCAAA-3' |
| GFAP | F: 5′-CTTGACCTGCGACCTTGAGT-3' |
|  | R: 5′-TTTCTTCGCCCTCCAGCAAT-3' |
| S100β | F: 5'-GAAACTCTGACCCCGTTCCC-3' |
|  | R: 5'-AGAAAGCTGTCCAACGGAGG-3' |
| GAPDH | F: 5'-AGTGCCAGCCTCGTCTCATA-3' |
|  | R: 5'-GATGGTGATGGGTTTCCCGT-3' |

*Note: RT-qPCR, reverse transcription-quantitative polymerase chain reaction; F, forward; R, reverse; STC-1, stanniocalcin-1; ROS, reactive oxygen species; NF-κB, nuclear factor κB; GluR1, glutamate receptor 1; BNDF, brain-derived neurotrophic factor; GFAP, glial fibrillary acidic protein; GAPDH, glyceraldehyde-3-phosphate dehydrogenase.*
